# Endotheliopathy syndromes, TA-TMA, and SOS, are risk factors for morbidity and mortality in critically ill pediatric hematopoietic cell transplant recipients

**DOI:** 10.3389/fonc.2025.1642939

**Published:** 2025-09-03

**Authors:** Shivani Goel, Erin Frost, Keiko Tarquinio, Pradip Kamat, Taylor Fitch, Elizabeth Stenger, Katie Liu, Muna Qayed, Zhulin He, Adrianna Westbrook, Kirsten M. Williams, Michelle L. Schoettler

**Affiliations:** ^1^ Department of Pediatrics, Division of Critical Care Medicine, Children’s Healthcare of Atlanta, Atlanta, GA, United States; ^2^ Aflac Cancer and Blood Disorders Center, Department of Pediatrics, Division of Hematology and Oncology, Children’s Healthcare of Atlanta, Emory University School of Medicine, Atlanta, GA, United States; ^3^ Department of Biostatistics, Children’s Healthcare of Atlanta, Emory University School of Medicine, Atlanta, GA, United States

**Keywords:** pediatric, hematopoietic cell therapy, intubation, mechanical ventilation, outcomes, sinusoidal obstructive syndrome (SOS), transplant associated thrombotic microangiopathy (TA-TMA), endotheliopathy syndrome

## Abstract

**Background:**

Pediatric hematopoietic cell transplant (HCT) recipients who require intensive care unit (PICU) admission historically have high mortality rates. The HCT landscape is rapidly changing with the incorporation of novel graft versus host disease (GVHD), infection prevention strategies, and diagnosis and treatment of endothelial disorders—all potentially impacting the risk factors for morbidity and outcomes of critically ill pediatric HCT recipients.

**Methods:**

This IRB-approved single-center, retrospective cohort included all allogeneic recipients from 2019 to 2023 who required ICU admission in the first year post-HCT.

**Results:**

A total of 91 unique PICU admissions in 56 HCT patients were identified. The median age at HCT was 8.4 years; 30 (54%) were female. Moreover, 34 (61%) developed early endotheliopathy syndrome: 27 (48.2%) TA-TMA (all treated with eculizumab), 21 (37.5%) SOS (all treated with defibrotide), and 14 (25%) both TA-TMA and SOS. A total of 40 admissions (44%) required IMV. The risk factors (RF) for IMV included younger age, TA-TMA, SOS, RRT, and PICU length of stay ≥14 days. Of those requiring IMV, 15 patients (37.5%) failed extubation; no HCT or clinical features predicted extubation failure. Furthermore, 23 admissions (25.3%) required renal replacement therapy (RRT). The RF for RRT included TA-TMA, SOS, PICU LOS, and weight gain of ≥5% from dry weight at the time of PICU admission. The duration that weight exceeded 10% of the dry weight before RRT was associated with the inability to come off RRT. The 100-day PICU-related mortality was 25% (95% CI: 14–37), though the 1-year NRM from first ICU admission was 41% (95% CI: 31–51). RF for non-relapse-related mortality (NRM) included TA-TMA and required RRT. Grade 3–4 acute GVHD was not a risk factor for ICU morbidity nor mortality. Infection was also not a risk factor, but the very high proportion of infection in the cohort limits the analysis.

**Discussion:**

In this contemporary cohort with a high prevalence of infection, the NRM of critically ill allogeneic HCT recipients was lower than the historic rates, and 62.5% of children requiring IMV were successfully extubated. SOS and TA-TMA were risk factors for highly morbid ICU complications and death despite early intervention. Alternative approaches to these diseases and their drivers and initiation of early RRT may avert death.

## Introduction

Hematopoietic cell transplantation (HCT) offers curative therapy for children with malignant and non-malignant diseases (1). However, toxicities, including the development of organ failure, are a major barrier to HCT success and account for ~33% of early deaths post-HCT (<100 days) (2, 3). Approximately 15%–40% of pediatric HCT recipients require intensive care unit (PICU) admission and support. Previously described risk factors for PICU admission in HCT patients include allogeneic HCT, HCT indication including inborn errors of metabolism, and severe acute GVHD (2, 4, 5). Historically, the mortality rates of critically ill HCT recipients have exceeded 50% (6–8). Prior described risk factors for death in this cohort include respiratory failure requiring invasive mechanical ventilation (IMV), severe acute graft versus host disease (GVHD), longer duration of non-invasive positive pressure (NIPPV) prior to IMV, fluid overload, and renal failure requiring renal replacement therapy (RRT) (9–12).

While these prior risk factors and poor outcomes were evident over several decades, contemporary HCT practices are rapidly changing. The adoption of novel GVHD prophylaxis approaches including post-transplant cyclophosphamide and abatacept (13–15), infection prophylaxis strategies including cytomegalovirus prophylaxis (16), diagnostic criteria for sinusoidal obstructive syndrome (17), use of prophylactic defibrotide (18, 19), and novel diagnostic, risk stratification, and early intervention approaches of transplant-associated thrombotic microangiopathy (TA-TMA) (20, 21) could all alter the previously identified risk factors among allogeneic HCT recipients. A recent study demonstrated that prolonged non-invasive ventilation led to high rates of intubation, peri-intubation arrest, and mortality, suggesting that early IMV may be beneficial in this particularly vulnerable population (6). These findings and possible practice-changing approaches highlight a novel endpoint, successful extubation from IMV and risk factors for IMV failure, for which data are scant.

We hypothesized that improvements in supportive care and implementation of newer approaches have led to improved outcomes in critically ill pediatric HCT recipients and altered the previously identified risk factors for PICU-associated morbidity and death. The objective of this study was to describe the risk factors and outcomes of ICU events in allogeneic HCT patients requiring PICU care, including IMV, IMV extubation failure, RRT, and mortality.

## Patients and methods

### Data source

In this Institutional Review Board-approved, single-center, retrospective single study, data were extracted from the electronic medical record (EMR) of consecutive allogeneic HCT recipients who received an HCT between April 2019 and December 2023 and were admitted to the PICU within 1 year of HCT. The patients were identified using an internal HCT database. Data extracted included patient and transplant characteristics, unique PICU admission(s), PICU course(s), and survival. All TA-TMA, SOS, acute GVHD, and causes of death were adjudicated by authors MLS, KMW, and PK in addition to 20% of random data fields. Any discrepancies in data were validated by additional members of the research team prior to analysis. Patients were excluded from the analysis if they had a do not resuscitate (DNR) or do not intubate (DNI) order in place at the time of PICU admission.

### Outcome measures/diagnostic criteria/definitions

Primary endpoints included IMV, IMV extubation failure, PICU-related mortality, and non-relapse-related mortality (NRM). Failed extubation from IMV was defined as reintubation within 72 h of extubation or death prior to an extubation attempt. PICU-related mortality was defined as death during the first PICU admission. NRM was defined as death of any cause other than relapse, which was treated as a competing risk. Sinusoidal obstruction syndrome (SOS) was diagnosed using European Bone Marrow Transplant criteria (17). Transplant-associated thrombotic microangiopathy (TA-TMA) was diagnosed using Jodele criteria (22), though all patients also met later published consensus criteria (20). Patients with either TA-TMA or SOS were considered to have an “early endotheliopathy syndrome”. Acute GVHD (aGVHD) was graded and staged using MAGIC criteria (23). Acute kidney injury (AKI) was defined as an increase in creatinine by >0.3 mg/dL in 48 h or an increase in serum creatinine >1.5 times against baseline values per KDIGO guidelines (24). Fluid overload was defined as weight gain from dry weight to time of ICU admission, and in those who required renal replacement, weight at the time of RRT initiation. Dry weight is defined per institutional policy. Early post-HCT, the day 0 weight is considered the dry weight. Patients readmitted or admitted to the PICU later in their HCT course have an expected dry weight calculated by the nutritionists, which accounts for expected growth. Infections that occurred during any PICU admission(s) were captured. Infections were identified via positive culture (bacteria, virus, or fungus) in blood and tissue culture and PCR testing. Next-generation sequencing (Karius^®^) tests were also included if the clinical team used these data to determine treatments.

The indication for PICU admission was abstracted from documentation at the time of transfer. Etiology of respiratory failure, if present, was abstracted from the indication listed in the intubation procedure documentation. Primary and secondary causes of death, where applicable, were abstracted from the death summary and autopsy if available, and attribution was assigned using previously described methods (25).

### Statistical analysis

Descriptive statistics were used to summarize patient characteristics. Fishers exact test and Wilcoxon rank sum test were used for between-group comparisons in the analysis of categorical and continuous variables, respectively. Mixed models were used to assess risk factors for IMV, RRT, and death, which account for repeated ICU admissions by treating patients as a random effect and reporting robust standard errors for hypothesis testing (26). Logistic regression models were used to determine risk factors for the first IMV extubation failure. TA-TMA, SOS, endotheliopathy syndromes, acute GVHD, IMV, and RRT were treated as time-dependent variables in all models. Due to sample size restrictions and multiple co-linear variables, a multivariable analysis was not performed. Survival from IMV, RRT, and PICU were estimated from the day of first ICU admission using Kaplan–Meier methods. NRM was estimated from the day of first ICU admission treating relapse as a competing risk. Statistical significance was defined as *p <*0.05. R (v.4.3.2) was used for all statistical analyses.

## Results

### Patient and transplant characteristics

A total of 91 unique PICU admissions in 56 HCT patients were identified in the study period. The median age at HCT was 8.4 years (IQR, 2.6 to 15.1 years); 30 (54%) were female, 25 (45%) were White non-Hispanic, and 4 (7.1%) received second HCT prior to ICU admission. The most common indication for HCT was hematologic malignancy (*n* = 32, 57%), followed by immune deficiency/dysregulation (*n* = 14, 25%). Most patients received a bone marrow cell source (*n* = 37, 66%), an unrelated donor (*n* = 36, 64%), and an HLA match 8/8 (*n* = 39, 78%, **Table 1**). A total of 16 (29%) patients had a maximum acute GVHD grade 1–2 and 18 (32%) had grade 3 - 4.

**Table 1:** Demographics and HCT characteristics.

Characteristic	*N* = 56 *N* (%)
Age at HCT (median, IQR)	8.4 (2.6, 15.1)
Sex	
Female	30 (54%)
Male	26 (46%)
Race	
Black	13 (23%)
Other	9 (16%)
White	34 (61%)
Ethnicity	
Hispanic	9 (16%)
Non-Hispanic	47 (84%)
HCT number	
1	52 (93%)
2	4 (7.1%)
Transplant indications	
Hematologic malignancy	32 (57%)
Hematologic non-malignancy	7 (13%)
Immunologic	14 (25%)
Metabolic disease	3 (5.4%)
Conditioning regimen	
Myeloablative	41 (73%)
Reduced toxicity/intensity	15 (27%)
Source of transplant	
Bone marrow	37 (66%)
Cord blood	2 (3.6%)
PBSC	17 (30%)
Matched related donor	
Related	20 (36%)
Unrelated	36 (64%)
HLA match	
8/8	39 (71%)
7/8	11 (20%)
≤6/8	5 (9.1%)
SOS	21 (38%)
Defibrotide treatment^a^	21 (38%)
Defibrotide prophylaxis	5 (8.9%)
TA-TMA	27 (48%)
Maximum acute GVHD	
Grade 0	22 (39%)
Grade 1–2	16 (29%)
Grade 3–4	18 (32%)
Infection	45 (80%)
Primary graft failure	3 (5.4)
Engraftment by time of PICU admission	28 (50%)
Days between neutrophil engraftment and PICU admission (median, IQR)	69 (39, 186)

a Of those who received defibrotide for SOS treatment, five were already on defibrotide for prophylaxis, but the course was extended for treatment.

SOS, sinusoidal obstruction syndrome; TA-TMA, transplant-associated thrombotic microangiopathy; GVHD, graft versus host disease; PBSC, peripheral blood stem cell.

### Early endothelial complications

**Figure 1:**
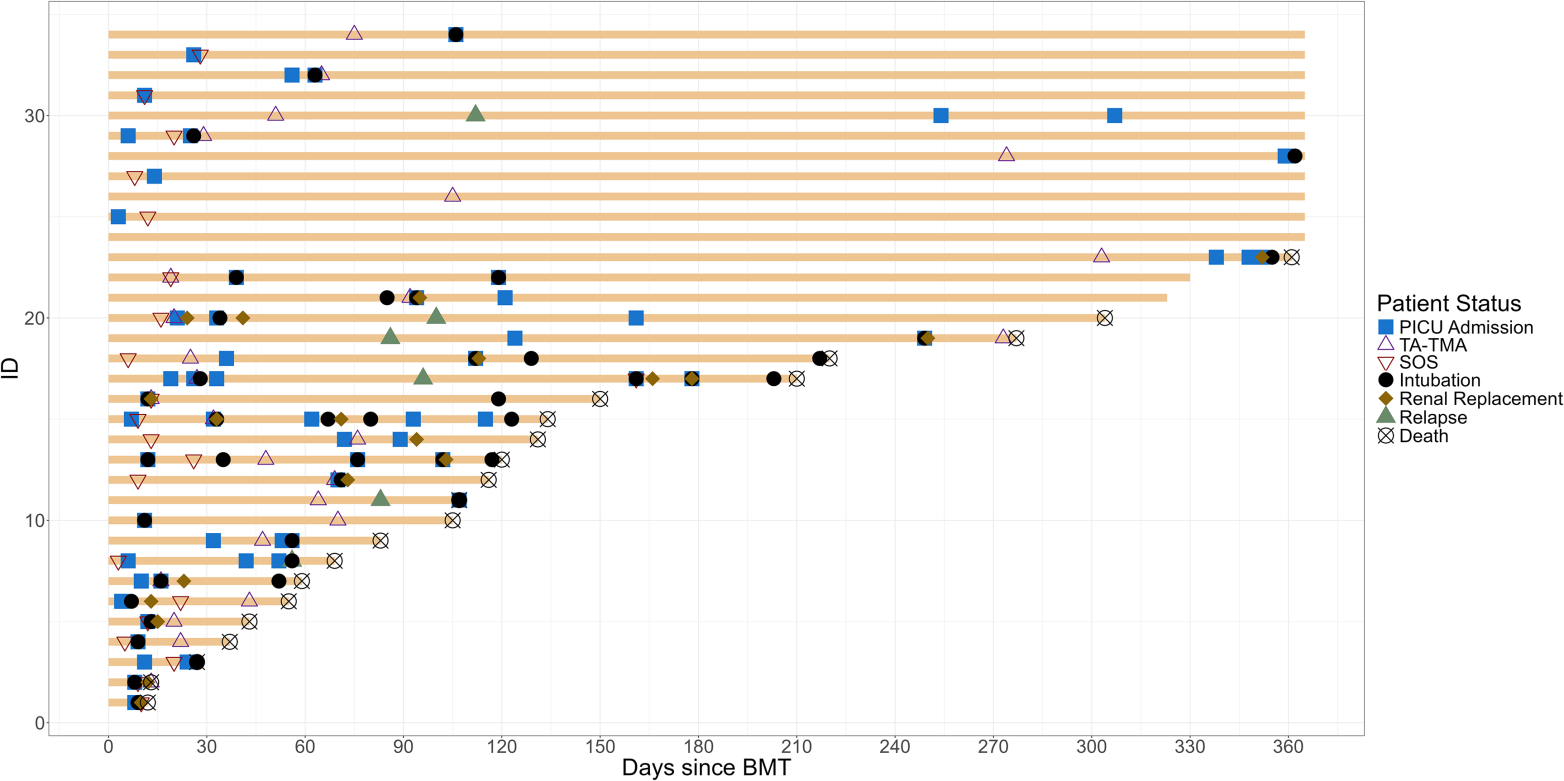
Swimmer plot of children with endotheliopathy syndrome. The clinical course from HCT to 1-year post-HCT of all patients with endotheliopathy syndrome is summarized. TA-TMA, transplant-associated thrombotic microangiopathy; SOS, sinusoidal obstructive syndrome; PICU, pediatric intensive care unit.

There were 34 (61%) patients who had an early endotheliopathy syndrome. Twenty-seven (48%) had a diagnosis of TA-TMA, which occurred at a median of 47.5 days post-HCT (IQR, 22.8 to 73.8). All received TA-TMA-directed therapy with eculizumab; complement inhibition was achieved and confirmed using total complement and soluble C5b-9. Twenty one (38%) developed SOS at a median of 12 days post-HCT (IQR, 9 to 20). All were treated with defibrotide. All patients received ursodiol prophylaxis, and five (24%) received defibrotide prophylaxis. Among the five who received defibrotide prophylaxis, all developed SOS, though they had known SOS risk factors including osteopetrosis (*n* = 2), liver failure prior to HCT (*n* = 1), and inotuzumab prior to HCT (*n* = 2). In addition, 14 (25%) patients had both SOS and TA-TMA (**Figure 1**).

### Infections

A total of 66 (72.5%) admissions were complicated by one or more identified infections; many patients had more than one organism/infection and site involved. Viral infections were the most common (*n* = 54), with most detected in the blood (*n* = 26), nasopharyngeal swab (*n* = 14), and lungs via bronchoscopy (*n* = 3). The most frequently identified viral infections included adenovirus (*n* = 11), human polyoma virus 1 (BK virus, *n* = 8), and cytomegalovirus (*n* = 7). Among bacterial infections (*n* = 40), the majority were detected in the blood (*n* = 27), gut (*n* = 4), and lungs (*n* = 3). The most frequently identified organisms included *Staphylococcus* (*n* = 13), *Streptococcus* (*n* = 4), and *Clostridium difficile* (*n* = 4). Fungal infections were the least common (*n* = 6), though *Aspergillus* was identified in the lungs (*n* = 3) and CNS (*n* = 1) and *Candida* in the blood (*n* = 2, **Supplementary Figure S1**).

### ICU characteristics

There were 34 patients admitted to the PICU once, 14 patients twice, and eight patients ≥3 times. Re-admissions were for the same indication in nine (41%) patients. The median time from HCT to the first PICU admission was 33 days (IQR, 9.8 to 96). The median length of stay of the first PICU admission was 3.4 days (IQR, 1.7 to 8.3), and the median total length of all PICU stays was 7.3 days (IQR, 3 to 28.3). Increased work of breathing or hypoxia was the most common indication for PICU admission (*n* = 47, 51.6%), followed by hypotension (*n* = 18, 19.8%) and neurologic change (*n* = 13, 14.3%). A diagnosis of renal injury was common—37 (40.7%) admissions had documented AKI prior to PICU admission, and 23 (25.3%) required RRT. Vasopressors were required in 28 admissions (30.8%%), and eight (8.8%) ICU admissions were complicated by cardiac arrest.

### Characteristics and risk factors for intubation and mechanical ventilation

Of the 91 PICU admissions, 40 (44%) required intubation and mechanical ventilation (IMV). Increased work of breathing or hypoxia was the most common indication for ICU admission in those requiring IMV (*n* = 29, 73%), followed by altered mental status (*n* = 4, 10%). One patient was initially intubated for procedural sedation but remained in the PICU due to the development of post-operative complications requiring ongoing IMV. The median time from PICU admission to the first IMV was 0 days (IQR, 0 to 1), and it occurred with a median of 54 days from HCT (IQR, 14 to 104 days). Prior to IMV, 20 (50%) patients were supported with high-flow nasal cannula (HFNC) for a median of 13.5 h (IQR, 5.7 to 31.7). Moreover, 10 (25%) required non-invasive positive pressure ventilation (NIPVV) and received this support for a median of 5.9 h (IQR, 2.9 to 27.1) prior to IMV.

On univariable analysis, the risk factors for IMV included TA-TMA (HR 2.58, 95% CI: 1.39–4.77), SOS (HR 2.08, 95% CI: 1.12–3.87), RRT (HR 4.79, 95% CI: 2.7–8.49), and PICU LOS ≥14 days (ref: <5 days, HR 6.55, 95% CI: 2.94–14.58). Age at time of PICU admission was also a risk for IMV—for every increase in 1 year of age, the HR of IMV was 0.95 (95% CI: 0.9–0.99); thus, the risk of IMV was highest in younger patients. Grade 3–4 acute GVHD, infections, and day post-HCT of admission to the PICU were not risk factors for IMV (**Table 2**).

**Table 2:** Risk factors for intubation and mechanical ventilation.

Characteristic	No intubation, *N* = 51	Intubation, *N* = 40	HR (95% CI)	*P*-value
Age at admission	10.3 (2.4, 15.9)	3.7 (1.6, 10.6)	**0.95 (0.9, 0.99)**	**0.02**
SOS	11 (22%)	19 (48%)	**2.08 (1.12, 3.87)**	**0.02**
Grade 3–4 acute GVHD^a^	19 (37%)	17 (43%)	1.16 (0.61, 2.19)	0.65
TA-TMA	13 (25%)	23 (58%)	**2.58 (1.39, 4.77)**	**0.003**
RRT	2 (3.9%)	21 (53%)	**4.79 (2.7, 8.49)**	**<0.001**
Infection	36 (71%)	30 (75%)	1.36 (0.74, 2.51)	0.32
Relapse	15 (29%)	9 (23%)	0.74 (0.36, 1.53)	0.42
Graft failure	3 (5.9%)	0 (0%)	–	
HCT day at admission	56 (11, 179)	54 (14, 104)	1.00 (0.99, 1.00)	0.09
PICU LOS (days)				
<5 days	38 (75%)	12 (30%)	Ref	
≥14 days	2 (3.9%)	20 (50%)	**6.55 (2.94, 14.58)**	**<0.001**
5–14 days	11 (22%)	8 (20%)	1.93 (0.75, 4.91)	0.17
PICU indication^b^				
Hypotension	15 (29%)	3 (7.5%)		
Increased WOB/hypoxia	18 (35%)	29 (73%)		
Neurologic (AMS, seizure)	9 (18%)	4 (10%)		
Other	8 (16%)	3 (7.5%)		
Procedural	1 (2.0%)	1 (2.5%)		
HFNC	20 (39%)	20 (50%)	1.28 (0.71, 2.29)	0.41
NIPPV	7 (14%)	10 (25%)	1.36 (0.72, 2.58)	0.34
Weight change (category)				
<-5%	4 (7.8%)	0 (0%)	–	
-5% to 0% (0% not included)	12 (24%)	8 (20%)	0.97 (0.42, 2.25)	0.95
0% to 5% (5% not included)	23 (45%)	15 (38%)	Ref	
≥5%	12 (24%)	17 (43%)	1.73 (0.87, 3.45)	0.12
Transplant indications				
Hematologic malignancy	35 (69%)	21 (53%)		
Hematologic non-malignancy	3 (5.9%)	4 (10%)		
Immunologic	12 (24%)	12 (30%)		
Metabolic	1 (2.0%)	3 (7.5%)		
Engraftment by time of PICU admission	31 (61%)	30 (75%)	1.57 (0.78, 3.16)	0.20

SOS, sinusoidal obstruction syndrome; TA-TMA, transplant-associated thrombotic microangiopathy; GVHD, graft versus host disease; PBSC, peripheral blood stem cell; RRT, renal replacement therapy; LOS, length of stay; PICU, pediatric intensive care unit; WOB, work of breathing; AMS, altered mental status; HFNC, high-flow nasal cannula; NIPPV, non-invasive positive pressure ventilation. Bolded values indicate statistically significant risk factors.

a Maximum acute GVHD grade.

b Due to no exposure in one or more comparison groups, the hazard ratio could not be reliably estimated.

**Figure 2:**
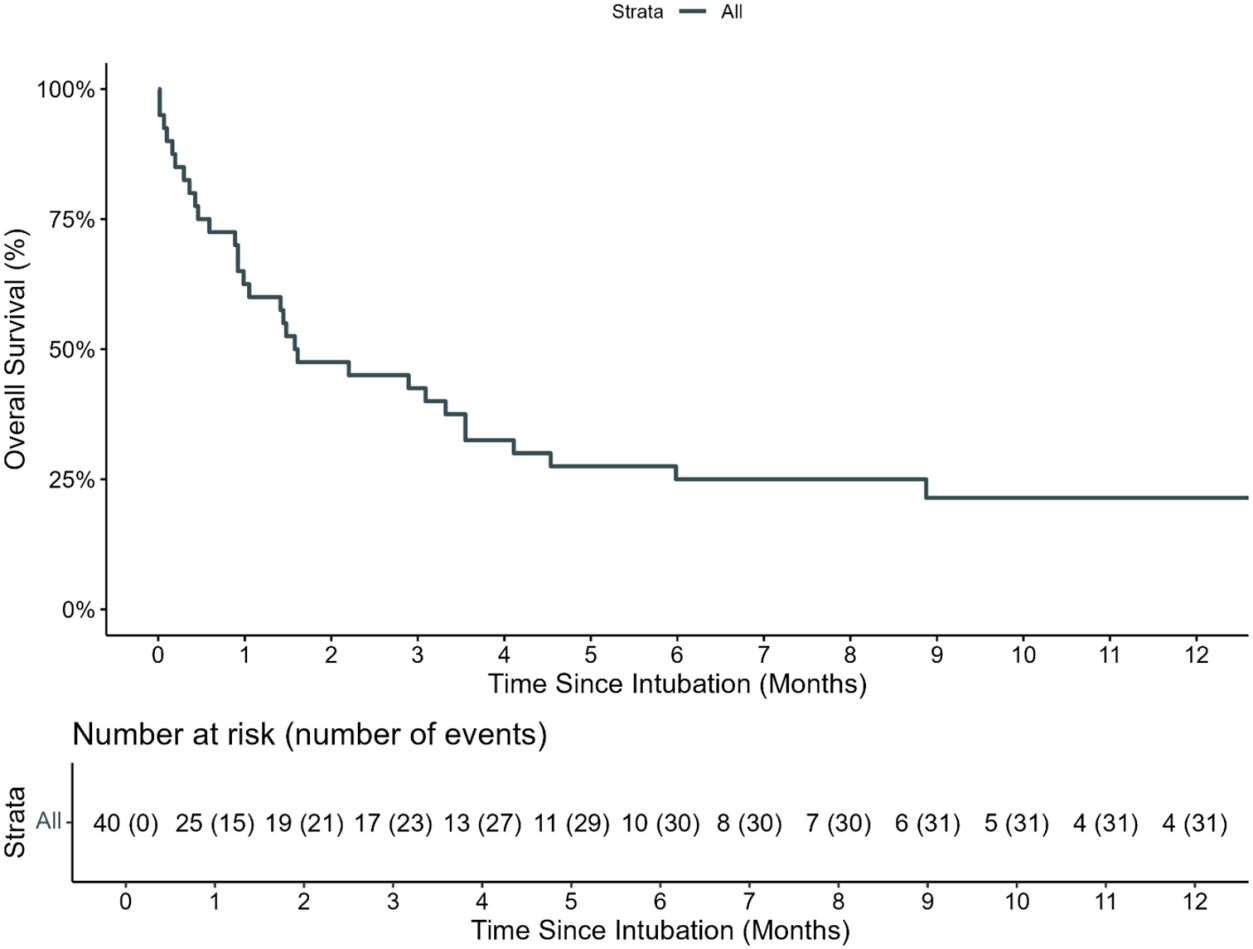
Overall survival of pediatric HCT recipients requiring IMV. The estimated 100-day and 1-year OS of pediatric HCT recipients who required intubation and mechanical ventilation (IMV) from day of first MIV was 40.0% (95% CI: 27.7%–57.8%) and 21.4% (95% CI: 10.6%–43.1%), respectively.

The estimated 100-day survival from IMV was 40% (95% CI: 27.7–57.8, **Figure 2**). A total of 25 (62.5%) IMV episodes resulted in a successful extubation on the first attempt. Of the 15 (37.5%) with failed first IMV extubation attempt, 12 (80%) died without attempting extubation, and three (20%) required re-intubation within 72 h. Among the three patients re-intubated within 72 h, all had successfully passed a prior CPAP trial. One patient was later successfully extubated and survived to PICU discharge. There were no statistically significant risk factors for first IMV extubation failure (**Supplementary Table S1**).

### Cardiac arrests

Eight patients (14.3%) had at least one cardiac arrest during their individual admission. Five had a cardiac arrest during intubation, with return of spontaneous circulation achieved in all, though none survived to ICU discharge. Of the two patients who received NIPPV prior to intubation and cardiac arrest, both received NIPPV for <6 h prior to intubation. No patient received ECMO. The estimated 100-day survival from first cardiac arrest was (12.5%, 95% CI: 2.0–78.2%, **Supplementary Figure S2**). However, this single survivor died shortly after day 100, and there were no long-term survivors.

### Characteristics and risk factors for renal injury and renal replacement therapy

Acute kidney injury (AKI) was present at the time of PICU admission in 37 patients (40.7%). Furthermore, 23 (25.3%) PICU admissions required RR; among them, there were 25 discrete courses of RRT. The risk factors for requiring RRT included TA-TMA (HR 3.95, 95% CI: 1.78–8.75), SOS (HR 3.54, 95% CI: 1.57–7.98), PICU LOS 5–14 days (ref: <5 days, HR 7.16, 95% CI: 1.33–38.7), PICU LOS ≥14 days (ref: <5 days, HR 28.07, 95% CI: 6.51–121.08), requiring NIPPV (HR 2.74, 95% CI: 1.3–5.76), and weight gain of ≥5% from dry weight (HR 2.6, 95% CI: 1.07–6.3, **Table 3**). A total of 21 patients placed on RRT also required IMV; 20 required IMV prior to RRT and one after RRT. RRT was initiated at a median of 1.5 days following ICU admission (IQR, 1 to 4.3 days). The patients had a median increase of 6.8% in weight from dry weight at the time of RRT initiation (IQR, 1.7 to 18.4%). Of those who gained 5%–10% of body weight prior to RRT (*n* = 15, 60%), the patients’ weight exceeded ≥5% of the baseline at a median of 8 days (IQR, 5 to 16 days). Of those who gained >10% of baseline weight before RRT was initiated (*n* = 9, 36%), the median number of days that the patients had ≥10% weight gain was 7 days (IQR, 5.5 to 13 days). The patients remained on RRT for a median of 14 days (IQR, 6.3 to 28 days).

**Table 3:** Risk factors for renal replacement therapy.

Characteristic	No RR, *N* = 68	RR, *N* = 23	HR (95% CI)	*P*-value
Age at admission	6.7 (2.4, 15.5)	6.1 (1.3, 13.9)	0.97 (0.92, 1.03)	0.34
SOS	17 (25%)	13 (57%)	**3.54 (1.57, 7.98)**	**0.002**
Grade 3–4 acute GVHD^a^	27 (40%)	9 (39%)	0.86 (0.37, 1.99)	0.72
TA-TMA	20 (29%)	16 (70%)	**3.95 (1.78, 8.75)**	**<0.001**
Infection	47 (69%)	19 (83%)	1.93 (0.61, 6.1)	0.26
Relapse	18 (26%)	6 (26%)	1.01 (0.44, 2.33)	0.99
Graft failure	3 (4.4%)	0 (0%)	–	
HSCT day at admission	56 (13, 123)	48 (12, 112)	1.00 (1.00, 1.00)	0.76
PICU LOS (days)				
<5 days	48 (71%)	2 (8.7%)	Ref	
5–14 days	14 (21%)	5 (22%)	**7.16 (1.33, 38.7)**	**0.02**
≥14 days	6 (8.8%)	16 (70%)	**28.07 (6.51, 121.08)**	**<0.001**
PICU indication				
Hypotension	17 (25%)	1 (4.3%)		
Increased WOB/hypoxia	30 (44%)	17 (74%)		
Neurologic (AMS, seizure)	11 (16%)	2 (8.7%)		
Other	9 (13%)	2 (8.7%)		
Procedural	1 (1.5%)	1 (4.3%)		
Maximum ventilatory support				
None	28 (41%)	0 (0%)	Ref	
HFNC	15 (22%)	0 (0%)	–^b^	
NIPPV	6 (8.8%)	1 (4.3%)	–^b^	
IMV	19 (28%)	22 (96%)	–^b^	
Weight change (category)				
<-5%	4 (5.9%)	0 (0%)	–^b^	
-5% to 0% (0% not included)	16 (24%)	4 (17%)	1.03 (0.32, 3.31)	0.96
0% to 5% (5% not included)	31 (46%)	7 (30%)	Ref	
≥5%	17 (25%)	12 (52%)	**2.6 (1.07, 6.3)**	**0.03**
Transplant indications				
Hematologic malignancy	40 (59%)	16 (70%)		
Hematologic non-malignancy	6 (8.8%)	1 (4.3%)		
Immunologic	18 (26%)	6 (26%)		
Metabolic	4 (5.9%)	0 (0%)		
Had neutrophil engraftment by PICU admission	46 (68%)	15 (65%)	0.92 (0.42, 2.04)	0.84

SOS, sinusoidal obstruction syndrome; TA-TMA, transplant-associated thrombotic microangiopathy (TA-TMA); GVHD, graft versus host disease; PBSC, peripheral blood stem cell; RRT, renal replacement therapy (RRT); LOS, length of stay; PICU, pediatric intensive care unit; WOB, work of breathing; AMS, altered mental status; HFNC, high-flow nasal cannula; NIPPV, non-invasive positive pressure ventilation. Bolded values indicate statistically significant risk factors.

a Maximum acute GVHD grade.

^b^Due to no exposure in one or more comparison groups, the hazard ratio could not be reliably estimated.

**Figure 3:**
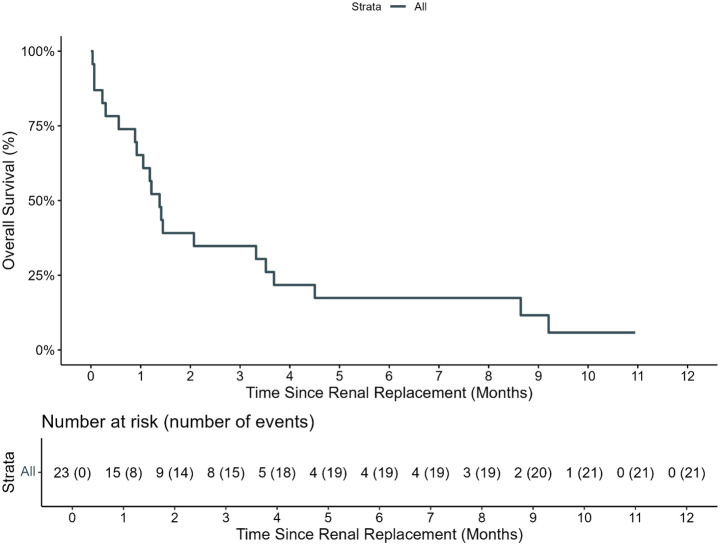
Overall survival of patients requiring renal replacement therapy. The estimated 100-day OS of pediatric HCT recipients who required renal replacement therapy (RRT) from day of first RRT was 34.8% (95% CI: 19.2%–62.9%).

There were 19 (82.6%) patients who died while on RRT, and three (13%) survived discharge from the PICU (**Supplementary Table S2**). One patient is alive but remains RRT dependent more than 2 years later, one died of relapsed leukemia, and one patient is alive with recovered renal function at the last follow-up. The number of days that the weight was >10% above dry weight at CRRT initiation was inversely associated with the successful cessation of RRT (0.98, 95% CI: 0.83–1.08). All patients who gained ≥10% of body weight prior to starting renal replacement died, though survival after RRT in the entire cohort was poor, with an estimated day-100 survival from initiating RRT of 34.8% (95% CI: 19.2–62.9, **Figure 3**).

### Characteristics and risk factors for NRM

**Figure 4:**
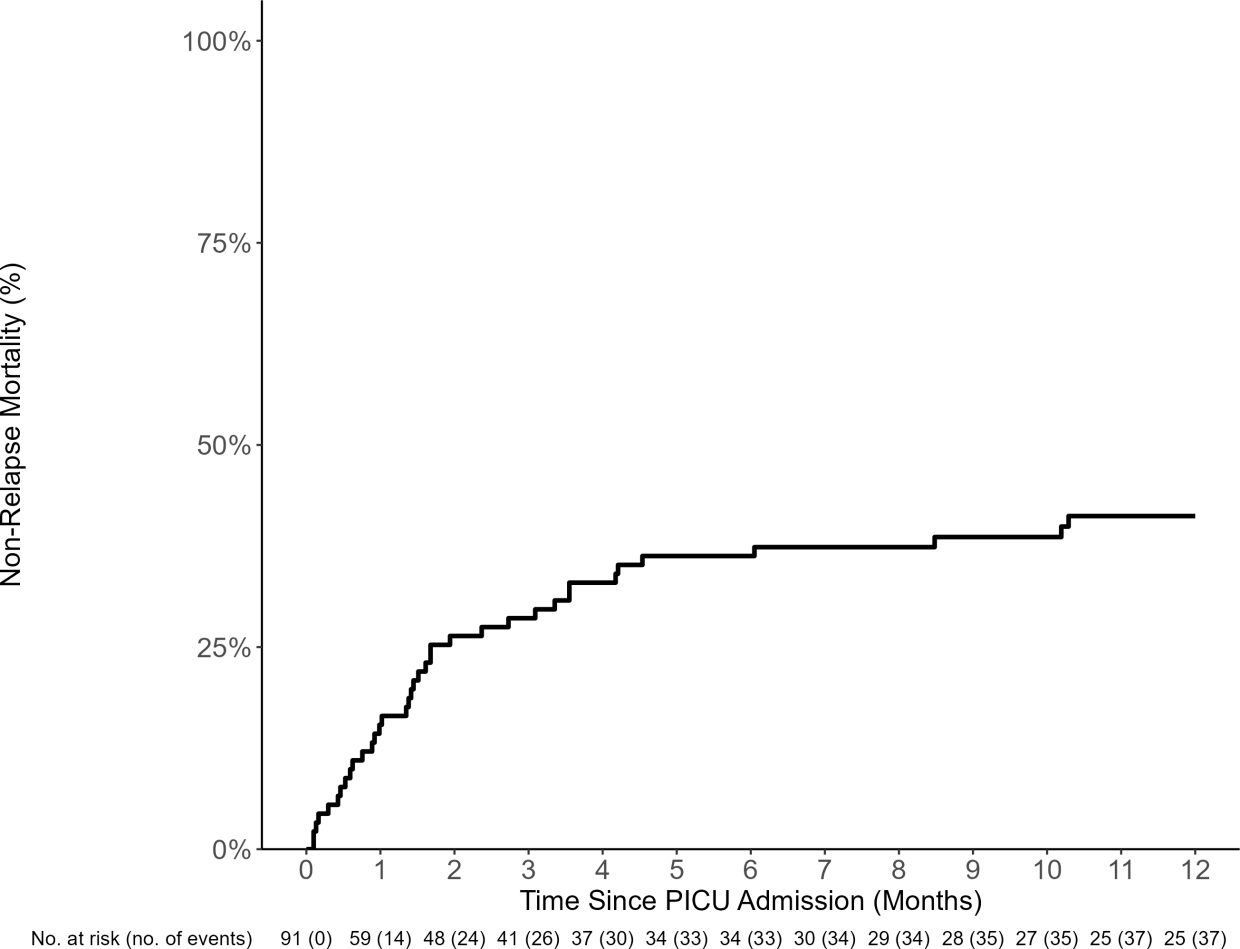
NRM of HCT recipients requiring PICU care (including all admissions). The estimated 100-day and 1-year PICU non-relapse-related mortality (NRM) from day of PICU admission was 30% (95% CI: 21%–39%) and 41% (95% CI: 31%–51%), respectively.

The estimated PICU mortality (death prior to the first PICU discharge) 100 days from the first PICU admission was 25% (95% CI: 14–37, **Supplementary Figure S3**). The median time to death from PICU admission in those who died during the first admission was 67 days (IQR, 31 to 138). However, the 1-year NRM from all ICU admission was 41% (95% CI: 31–51, **Figure 4**). The most common causes of death included organ failure (*n* = 17, 53.1%), infection (*n* = 20, 62.5%), and relapsed disease (*n* = 11, 35.5%, **Supplementary Table S3**). The univariable risk factors for NRM included TA-TMA (HR 4.41, 95% CI: 1.58–12.34) and RRT (HR 2.11, 95% CI: 1.07–4.15) (**Table 4**). Neither grade 3–4 acute GVHD nor infections were risk factors for NRM.

**Table 4:** Risk factors for non-relapsed related mortality (NRM).

Characteristic	Non-NRM, *N* = 53	NRM, *N* = 38	HR (95% CI)	*P*-value
Age at admission	6.8 (2.1, 15.8)	4.8 (1.3, 13.9)	0.97 (0.88, 1.08)	0.60
SOS	14 (26%)	16 (42%)	2.27 (0.94, 5.47)	0.07
Grade 3–4 acute GVHD^a^	21 (40%)	15 (39%)	1.25 (0.54, 2.88)	0.60
TA-TMA	16 (30%)	20 (53%)	**4.41 (1.58, 12.34)**	**0.005**
RRT	8 (15%)	15 (39%)	**2.11 (1.07, 4.15)**	**0.03**
Infection	37 (70%)	29 (76%)	0.51 (0.2, 1.29)	0.15
Graft failure	2 (3.8%)	1 (2.6%)	2.12 (0.27, 16.74)	0.48
HCT day at admission	59 (19, 124)	51 (12, 112)	1 (1, 1.01)	0.44
PICU LOS (days)				
<5 days	33 (62%)	17 (45%)	Ref	
5–14 days	13 (25%)	6 (16%)	1.37 (0.54, 3.5)	0.51
≥14 days	7 (13%)	15 (39%)	0.32 (0.1, 1.06)	0.06
PICU indication				
Hypotension	12 (23%)	6 (16%)		
Increased WOB/Hypoxia	23 (43%)	24 (63%)		
Neurologic (AMS, seizure)	9 (17%)	4 (11%)		
Other	9 (17%)	2 (5.3%)		
Procedural	0 (0%)	2 (5.3%)		
Maximum ventilatory support				
None	21 (40%)	7 (18%)	Ref	
HFNC	8 (15%)	7 (18%)	0.29 (0.07, 1.26)	0.10
NIPPV	5 (9.4%)	2 (5.3%)	0.73 (0.22, 2.46)	0.61
IMV	19 (36%)	22 (58%)	0.45 (0.15, 1.33)	0.15
Weight change (category)				
<-5%	3 (5.7%)	1 (2.6%)	2.12 (0.23, 19.16)	0.50
-5% to 0% (0% not included)	13 (25%)	7 (18%)	1.34 (0.52, 3.44)	0.54
0% to 5% (5% not included)	22 (42%)	16 (42%)	Ref	
≥ 5%	15 (28%)	14 (37%)	0.88 (0.3, 2.58)	0.81
Transplant indications				
Hematologic malignancy	35 (66%)	21 (55%)		
Hematologic non-malignancy	7 (13%)	0 (0%)		
Immunologic	11 (21%)	13 (34%)		
Metabolic	0 (0%)	4 (11%)		

SOS, sinusoidal obstruction syndrome; TA-TMA, transplant-associated thrombotic microangiopathy; GVHD, graft versus host disease; PBSC, peripheral blood stem cell; RRT, renal replacement therapy; LOS, length of stay; PICU, pediatric intensive care unit; WOB, work of breathing; AMS, altered mental status; HFNC, high-flow nasal cannula; NIPPV, non-invasive positive pressure ventilation. Bolded values indicate statistically significant risk factors.

a Maximum acute GVHD grade.

## Discussion

In a contemporary pediatric HCT cohort, the 100-day PICU mortality was low at 25%. This is in stark contrast from historical data in which ICU-associated mortality exceeded 60% (2, 27–29). However, many patients were re-admitted to the PICU and NRM in the cohort 1 year from the first PICU admission, increased to 41%, similar to other recent HCT studies (2). Nearly one-third of deaths in this critically ill cohort were ultimately due to relapsed disease as expected from national cohorts. Thus, we focused on reporting the risk factors for NRM. Previously reported risk factors, including RRT, remained associated with increased NRM in this study (2, 11, 30, 31). However, we also found that endotheliopathy syndromes, particularly TA-TMA and SOS, were risk factors for both ICU morbidity, mechanical ventilation and RRT, and NRM despite early treatment of both complications with best available therapy per expert consensus (eculizumab and defibrotide, respectively) (32, 33). Given that endotheliopathy syndromes contribute to leaky vessels that compromise pulmonary function and renal injury, it is not surprising that this comorbidity was linked to requiring increased respiratory and renal support. All patients received SOS prophylaxis with urosodiol, and five (24%) patients received defibrotide prophylaxis. Our center has a standard approach to only offer defibrotide prophylaxis to the highest-risk patients. While all patients on prophylaxis developed SOS, this cohort was enriched for risk factors including osteopetrosis as an HCT indication and prior liver failure. An early alternative treatment of early endothelial complications may offer an opportunity to avert some organ failure after HCT, leading to even greater improvements in survival.

Respiratory distress was the most common cause of PICU admission, and 44% of admissions required IMV. In this cohort, 62.5% of children were successfully extubated after requiring IMV—again demonstrating stark improvements compared to historical data (34). We were unable to identify the risk factors for extubation failure, perhaps due to sample size or cohort heterogeneity. Recent data indicate that the use of NIPPV for more than 6 h before intubation was associated with an increased risk of cardiac arrest and PICU mortality in the HCT population (6, 7, 9). Experts have subsequently proposed that early intubation may be beneficial in this cohort (6, 9). These studies were published during the study period and may have influenced the treatment strategies in this cohort. Notably, in contrast to prior studies, the patients who had a cardiac arrest at the time of intubation were supported with NIVV for less than 6 h prior to intubation. Also in contrast to prior studies, neither the use of HFNC nor NIVV was a statistically significant risk factor to require IMV, and IMV was not a risk factor for NRM. During the study period, our institutional practice has largely moved toward early intubation in recipients with significant cardiorespiratory disease, as opposed to trialing or escalating non-invasive support. This change in practice may have contributed to why neither HFNC nor NIPPV use was associated with IMV need and why IMV was not a risk factor for NRM. While additional studies are needed to determine whether adopting an early intubation approach impacts survival after HCT, these data and others support early intubation and the aggressive management of acute respiratory failure in HCT recipients.

Acute kidney injury is a well-described complication of pediatric HCT and is associated with increased mortality, particularly renal failure requiring RRT (2, 11, 27). In this contemporary cohort, 23% of PICU admissions required RRT, nearly twice the incidence reported in other cohorts (5, 27). Furthermore, survival was poor in this cohort with an estimated survival 100 days from RRT initiation of 34.8%. Similar cohorts have reported mortality rates after RRT ranging from 55% to 65% (35, 36). We attribute the high incidence of renal replacement in this cohort in part to the high incidence of endotheliopathy syndromes, SOS, and TA-TMA. Both diseases were risk factors for requiring RRT in this study, and TA-TMA is a described risk factor for RRT in prior studies (37, 38).

Prior studies have reported that early fluid overload is independently associated with PICU mortality (39, 40). While there are limited data on the impact of timing of initiation of RRT in this specific cohort, data suggest that early RRT is beneficial in other cohorts with renal failure (41, 42). In this cohort, 52% of patients who required RRT had evidence of fluid overload at the time of initiation (≥5% weight gain). Furthermore, all five patients with ≥10% increase in weight from baseline at the time of initiating RRT died. Among those with ≥10% weight, all had a diagnosis of SOS. Our data suggest that increased weight is a proxy for more severe renal injury. This may reflect that RRT was initiated later in the disease course that led to primary (e.g., TA-TMA) or secondary renal injury (e.g., septic shock), decreasing the likelihood of salvaging renal function. More studies are needed to determine if early RRT in critically ill HCT recipients could improve the outcomes. However, our data add to a body of literature supporting vigilant fluid management post-HCT with liberal use of diuretics and early RRT in critically ill HCT recipients who are refractory to diuresis (43–45).

It was unexpected that grade 3–4 acute GVHD was not a risk factor for IMV, extubation failure, nor death. While improvements in acute GVHD prevention could have decreased the incidence of this complication, our population was more enriched for aGVHD than other larger studies (5, 27). Survival after severe GVHD is improving over time, perhaps due to novel agents to treat severe and refractory GVHD (46). Improved survival and outcomes in this cohort may result in the diminished risk for ICU-related morbidity and mortality. It is also possible that aGVHD remained a risk factor for IMV, extubation failure, and death, but our small sample size impeded our ability to detect a true association. In contrast to a prior study (47), neutrophil engraftment was not a risk factor for IMV or IMV extubation failure. Most patients (*n* = 30, 75%) in this study had neutrophil engraftment at the time of first IMV, perhaps precluding this analysis.

While infections were not identified as a risk for poor outcomes in our analysis, the high prevalence in the cohort limits this statistical analysis. Furthermore, to ensure reproducibility, we included only patients with documented infections. Patients with culture-negative septic shock and infections documented on imaging (i.e., chest computed tomography that demonstrates pneumonia) were not included in the analysis. Notably, the rate is similar to, though higher than, prior data in which the coincidence of infections with other co-morbidities was high in HCT recipients requiring ICU (5, 27). The high rate of infections in those admitted to the ICU in both studies and the high rate of infectious-related NRM in our data suggest that this is an important driver of morbidity in critically ill patients. Furthermore, the data suggest that we may not even be capturing all of the infections contributing to IMV; underdiagnosis of lung infections (and thus lack of treatment) has previously been linked to death in HCT recipients (48). It is possible that the infectious risks may be higher in our modern cohort due to impaired immunity in the setting of novel pre-HCT therapies (e.g., newer leukemia therapies, prior immune suppression in patients with immune dysregulation) and novel acute GVHD prophylaxis approaches. Since aGVHD and infections are major drivers of TA-TMA, a significant contributor to poor outcomes, it is probable that infections play a key role in vulnerable HCT recipients. In the future, studies designed to mitigate infectious risk could better evaluate the impact of infection on outcomes in critically ill pediatric HCT recipients.

There were several limitations to this study. This is a single-center, retrospective review with a limited sample size. However, we had robust, granular data of 91 PICU admissions in a diverse pediatric HCT cohort. Our institution previously had no clinical guidelines nor standard approaches for the escalation of respiratory support or RRT, contributing to heterogeneity in this study. Although many of our results aligned with those from large multi-center reports, additional future studies across multiple institutions are needed to validate our results.

In summary, we described in detail a contemporary cohort of HCT recipients who required ICU admission and identified endotheliopathy syndrome as a novel risk factor for IMV, RRT, and mortality in this cohort. Our data support that survival may be improving in those who require IMV. While outcomes were poor in those requiring RRT in this cohort, it is possible this is due to the late initiation of RRT in the setting of severe fluid overload and TA-TMA. The impact of early renal replacement and alternative TA-TMA-directed therapy bears additional investigation as does the role of infection. Additional multi-institutional studies are needed to validate these findings.

## Data Availability

The raw data supporting the conclusions of this article will be made available by the authors, without undue reservation.

## Supplementary material

Supplementary Table 1 Risk factors for first IMV extubation failure.

Supplementary Table 2 Outcomes of patients requiring renal replacement therapy by weight gain at time of RRT.

Supplementary Table 3 Causes of death (*n* = 32).

Supplementary Figure 1 Infections of HCT patients admitted to the ICU. Among the 56 patients and 91 ICU admissions, 66 admissions were complicated by ≥1 identified infection. Many patients had more than one organism/infection and site involved. Viral infections were the most common (*n* = 54), with most detected in the blood (*n* = 26), nasopharyngeal swab (*n* = 14), and lungs via bronchoscopy (*n* = 3). Among bacterial infections (*n* = 40), the majority were detected in the blood (*n* = 27), gut (*n* = 4), and lungs (*n* = 3). Fungal infections were the least common, though *Aspergillus* was identified in the lungs (*n* = 3) and CNS (*n* = 1) and *Candida* in the blood (*n* = 2).

Supplementary Figure 2 Overall survival in HCT patients admitted to the ICU who had a cardiac arrest. The estimated 100-day OS from the first cardiac arrest was 12.5% (95% CI: 2.0%–78.2%).

Supplementary Figure 3 PICU non-relapse mortality. The estimated 100-day PICU mortality (death during the first PICU admission from any cause other than relapse) from day of PICU admissions was 25% (95% CI: 14%–37%). Relapse was treated as a competing risk.

## References

[1] Phelan R Chen M Bupp C Bolon YT Broglie L Brunner-Grady J. Updated trends in hematopoietic cell transplantation in the United States with an additional focus on adolescent and young adult transplantation activity and outcomes. Transplant Cell Ther. (2022) 28:409.e1–409.e10. doi: 10.1016/j.jtct.2022.04.012, PMID: 35447374 PMC9840526

[2] Zinter MS Brazauskas R Strom J Chen S Bo-Subait S Sharma A. Intensive care risk and long-term outcomes in pediatric allogeneic hematopoietic cell transplant recipients. Blood Adv. (2024) 8:1002–17. doi: 10.1182/bloodadvances.2023011002, PMID: 38127268 PMC10879681

[3] Scarlata S Annibali O Santangelo S Tomarchio V Ferraro S Armiento D. Pulmonary complications and survival after autologous stem cell transplantation: predictive role of pulmonary function and pneumotoxic medications. Eur Respir J. (2017) 49. doi: 10.1183/13993003.01902-2016, PMID: 28356372

[4] Johnson AK Cornea S Goldfarb S Cao Q Heneghan JA Gupta AO. Risk factors predicting need for the pediatric intensive care unit (PICU) post-hematopoietic cell transplant, PICU utilization, and outcomes following HCT: a single center retrospective analysis. Front Pediatr. (2024) 12:1385153. doi: 10.3389/fped.2024.1385153, PMID: 38690520 PMC11059064

[5] Zinter MS Logan BR Fretham C Sapru A Abraham A Aljurf MD. Comprehensive prognostication in critically ill pediatric hematopoietic cell transplant patients: results from merging the center for international blood and marrow transplant research (CIBMTR) and virtual pediatric systems (VPS) registries. Biol Blood Marrow Transplant. (2020) 26:333–42. doi: 10.1016/j.bbmt.2019.09.027, PMID: 31563573 PMC6943183

[6] Rowan CM McArthur J Hsing DD Gertz SJ Smith LS Loomis A. Acute respiratory failure in pediatric hematopoietic cell transplantation: A multicenter study. Crit Care Med. (2018) 46:e967–74. doi: 10.1097/CCM.0000000000003277, PMID: 29965835

[7] Rowan CM Smith LS Loomis A McArthur J Gertz SJ Fitzgerald JC. Pediatric acute respiratory distress syndrome in pediatric allogeneic hematopoietic stem cell transplants: A multicenter study. Pediatr Crit Care Med. (2017) 18:304–9. doi: 10.1097/PCC.0000000000001061, PMID: 28178076

[8] Cater DT Fitzgerald JC Gertz SJ McArthur JA Daniel MC Mahadeo KM. Noninvasive ventilation exposure prior to intubation in pediatric hematopoietic cell transplant recipients. Respir Care. (2022) 67:1121–8. doi: 10.4187/respcare.09776, PMID: 35640999 PMC9994337

[9] Lindell RB Fitzgerald JC Rowan CM Flori HR Di Nardo M Napolitano N. The use and duration of preintubation respiratory support is associated with increased mortality in immunocompromised children with acute respiratory failure. Crit Care Med. (2022) 50:1127–37. doi: 10.1097/CCM.0000000000005535, PMID: 35275593 PMC9707852

[10] Gertz SJ Bhalla A Chima RS Emeriaud G Fitzgerald JC Hsing DD. Immunocompromised-associated pediatric acute respiratory distress syndrome: experience from the 2016/2017 pediatric acute respiratory distress syndrome incidence and epidemiology prospective cohort study. Pediatr Crit Care Med. (2024) 25:288–300. doi: 10.1097/PCC.0000000000003421, PMID: 38236083 PMC10994753

[11] Koh KN Sunkara A Kang G Sooter A Mulrooney DA Triplett B. Acute kidney injury in pediatric patients receiving allogeneic hematopoietic cell transplantation: incidence, risk factors, and outcomes. Biol Blood Marrow Transplant. (2018) 24:758–64. doi: 10.1016/j.bbmt.2017.11.021, PMID: 29196074

[12] Raymakers-Janssen P Lilien MR Tibboel D Kneyber MCJ Dijkstra S van Woensel JBM. Epidemiology and outcome of critically ill pediatric cancer and hematopoietic stem cell transplant patients requiring continuous renal replacement therapy: A retrospective nationwide cohort study. Crit Care Med. (2019) 47:e893–901. doi: 10.1097/CCM.0000000000003973, PMID: 31464768 PMC6798750

[13] Saliba RM Alousi AM Pidala J Arora M Spellman SR Hemmer MT. Characteristics of graft-versus-host disease (GvHD) after post-transplantation cyclophosphamide versus conventional gvHD prophylaxis. Transplant Cell Ther. (2022) 28 (10):681–93., PMID: 35853610 10.1016/j.jtct.2022.07.013 PMC10141544

[14] Watkins B Qayed M McCracken C Bratrude B Betz K Suessmuth Y. Phase II trial of costimulation blockade with abatacept for prevention of acute GVHD. J Clin Oncol. (2021) 39:1865–77. doi: 10.1200/JCO.20.01086, PMID: 33449816 PMC8260909

[15] de Witte MA Janssen A Nijssen K Karaiskaki F Swanenberg L van Rhenen A. αβ T-cell graft depletion for allogeneic HSCT in adults with hematological Malignancies. Blood Adv. (2021) 5:240–9. doi: 10.1182/bloodadvances.2020002444, PMID: 33570642 PMC7805311

[16] Marty FM Ljungman P Chemaly RF Maertens J Dadwal SS Duarte RF. Letermovir prophylaxis for cytomegalovirus in hematopoietic-cell transplantation. N Engl J Med. (2017) 377:2433–44. doi: 10.1056/NEJMoa1706640, PMID: 29211658

[17] Corbacioglu S Carreras E Ansari M Balduzzi A Cesaro S Dalle JH. Diagnosis and severity criteria for sinusoidal obstruction syndrome/veno-occlusive disease in pediatric patients: a new classification from the European society for blood and marrow transplantation. Bone Marrow Transplant. (2018) 53:138–45. doi: 10.1038/bmt.2017.161, PMID: 28759025 PMC5803572

[18] Corbacioglu S Cesaro S Faraci M Valteau-Couanet D Gruhn B Rovelli A. Defibrotide for prophylaxis of hepatic veno-occlusive disease in paediatric haemopoietic stem-cell transplantation: an open-label, phase 3, randomised controlled trial. Lancet. (2012) 379(9823):1301-9., PMID: 22364685 10.1016/S0140-6736(11)61938-7

[19] Mohty M Malard F Abecasis M Aerts E Alaskar AS Aljurf M. Prophylactic, preemptive, and curative treatment for sinusoidal obstruction syndrome/veno-occlusive disease in adult patients: a position statement from an international expert group. Bone Marrow Transplant. (2020) 55:485–95. doi: 10.1038/s41409-019-0705-z, PMID: 31576023 PMC7051913

[20] Schoettler ML Carreras E Cho B Dandoy CE Ho VT Jodele S. Harmonizing definitions for diagnostic criteria and prognostic assessment of transplantation-associated thrombotic microangiopathy: A report on behalf of the european society for blood and marrow transplantation, american society for transplantation and cellular therapy, asia-pacific blood and marrow transplantation group, and center for international blood and marrow transplant research. Transplant Cell Ther. (2023) 29:151–63. doi: 10.1016/j.jtct.2022.11.015, PMID: 36442770 PMC10119629

[21] Schoettler ML Patel S Bryson E Deeb L Watkins B Qayed M. Compassionate use narsoplimab for severe refractory transplantation-associated thrombotic microangiopathy in children. Transplant Cell Ther. (2024) 30:336.e1–8. doi: 10.1016/j.jtct.2023.12.017, PMID: 38145741 PMC11163410

[22] Jodele S Davies SM Lane A Khoury J Dandoy C Goebel J. Diagnostic and risk criteria for HSCT-associated thrombotic microangiopathy: a study in children and young adults. Blood. (2014) 124:645–53. doi: 10.1182/blood-2014-03-564997, PMID: 24876561 PMC4110664

[23] Major-Monfried H Renteria AS Pawarode A Reddy P Ayuk F Holler E. MAGIC biomarkers predict long-term outcomes for steroid-resistant acute GVHD. Blood. (2018) 131:2846–55. doi: 10.1182/blood-2018-01-822957, PMID: 29545329 PMC6014357

[24] Ostermann M Bellomo R Burdmann EA Doi K Endre ZH Goldstein SL. Controversies in acute kidney injury: conclusions from a Kidney Disease: Improving Global Outcomes (KDIGO) Conference. Kidney Int. (2020) 98:294–309. doi: 10.1016/j.kint.2020.04.020, PMID: 32709292 PMC8481001

[25] Copelan E Casper JT Carter SL van Burik JA Hurd D Mendizabal AM. A scheme for defining cause of death and its application in the T cell depletion trial. Biol Blood Marrow Transplant. (2007) 13:1469–76. doi: 10.1016/j.bbmt.2007.08.047, PMID: 18022577

[26] Austin PC. A tutorial on multilevel survival analysis: methods, models and applications. Int Stat Rev. (2017) 85:185–203. doi: 10.1111/insr.12214, PMID: 29307954 PMC5756088

[27] Zinter MS Dvorak CC Spicer A Cowan MJ Sapru A. New insights into multicenter PICU mortality among pediatric hematopoietic stem cell transplant patients. Crit Care Med. (2015) 43:1986–94. doi: 10.1097/CCM.0000000000001085, PMID: 26035280 PMC5253183

[28] Jenkins P Johnston LJ Pickham D Chang B Rizk N Tierney DK. Intensive care utilization for hematopoietic cell transplant recipients. Biol Blood Marrow Transplant. (2015) 21:2023–7. doi: 10.1016/j.bbmt.2015.07.026, PMID: 26238809

[29] Benz R Schanz U Maggiorini M Seebach JD Stussi G. Risk factors for ICU admission and ICU survival after allogeneic hematopoietic SCT. Bone Marrow Transplant. (2014) 49:62–5. doi: 10.1038/bmt.2013.141, PMID: 24056739

[30] Shorr AF Moores LK Edenfield WJ Christie RJ Fitzpatrick TM. Mechanical ventilation in hematopoietic stem cell transplantation: can We effectively predict outcomes? Chest. (1999) 116:1012–8., PMID: 10531167 10.1378/chest.116.4.1012

[31] Parikh CR McSweeney P Schrier RW. Acute renal failure independently predicts mortality after myeloablative allogeneic hematopoietic cell transplant. Kidney Int. (2005) 67:1999–2005. doi: 10.1111/j.1523-1755.2005.00301.x, PMID: 15840050

[32] Mahadeo KM Bajwa R Abdel-Azim H Lehmann LE Duncan C Zantek N. Diagnosis, grading, and treatment recommendations for children, adolescents, and young adults with sinusoidal obstructive syndrome: an international expert position statement. Lancet Haematology. (2020) 7:e61–72. doi: 10.1016/S2352-3026(19)30201-7, PMID: 31818728

[33] Jodele S Dandoy CE Aguayo-Hiraldo P Lane A Teusink-Cross A Sabulski A. A prospective multi-institutional study of eculizumab to treat high-risk stem cell transplantation–associated TMA. Blood. (2024) 143:1112–23. doi: 10.1182/blood.2023022526, PMID: 37946262 PMC10972707

[34] Paz HL Crilley P Weinar M Brodsky I. Outcome of patients requiring medical ICU admission following bone marrow transplantation. Chest. (1993) 104:527–31. doi: 10.1378/chest.104.2.527, PMID: 8339643

[35] Scales DC Thiruchelvam D Kiss A Sibbald WJ Redelmeier DA. Intensive care outcomes in bone marrow transplant recipients: a population-based cohort analysis. Crit Care. (2008) 12:R77. doi: 10.1186/cc6923, PMID: 18547422 PMC2481474

[36] Heyn M Ashcraft E Cheng C Epperly R Elbahlawan L. Continuous kidney replacement therapy in children with sinusoidal obstruction syndrome after hematopoietic cell transplant: outcome and liberation. Pediatr Blood Cancer. (2025) 72:e31473. doi: 10.1002/pbc.31473, PMID: 39654079 PMC11922561

[37] Epperla N Li A Logan B Fretham C Chhabra S Aljurf M. Incidence, risk factors for and outcomes of transplant-associated thrombotic microangiopathy. Br J Haematol. (2020) 189:1171–81. doi: 10.1111/bjh.16457, PMID: 32124435 PMC7726817

[38] Postalcioglu M Kim HT Obut F Yilmam OA Yang J Byun BC. Impact of thrombotic microangiopathy on renal outcomes and survival after hematopoietic stem cell transplantation. Biol Blood Marrow Transplant. (2018) 24:2344–53. doi: 10.1016/j.bbmt.2018.05.010, PMID: 29758394 PMC6230502

[39] Rondon-Clavo C Scordo M Hilden P Shah GL Cho C Maloy MA. Early fluid overload is associated with an increased risk of nonrelapse mortality after ex vivo CD34-selected allogeneic hematopoietic cell transplantation. Biol Blood Marrow Transplant. (2018) 24:2517–22. doi: 10.1016/j.bbmt.2018.07.031, PMID: 30055353 PMC6286243

[40] Elbahlawan L Qudeimat A Morrison R Schaller A. Fluid overload in children following hematopoietic cell transplant: A comprehensive review. J Clin Med. (2024) 13(21). doi: 10.3390/jcm13216348, PMID: 39518488 PMC11546381

[41] Seabra VF Balk EM Liangos O Sosa MA Cendoroglo M Jaber BL. Timing of renal replacement therapy initiation in acute renal failure: a meta-analysis. Am J Kidney Dis. (2008) 52:272–84. doi: 10.1053/j.ajkd.2008.02.371, PMID: 18562058

[42] Jiao R Lu X Liu M Zhu J Sun L Liu N. Randomized trial of early versus standard renal replacement therapy in patients with acute kidney injury after type A aortic dissection. Ann Thorac Surg. (2025). doi: 10.1016/j.athoracsur.2024.10.034, PMID: 39761941

[43] DiCarlo J Alexander SR. Acute kidney injury in pediatric stem cell transplant recipients. Semin Nephrol. (2008) 28:481–7. doi: 10.1016/j.semnephrol.2008.05.008, PMID: 18790368

[44] Raina R Abusin GA Vijayaraghavan P Auletta JJ Cabral L Hashem H. The role of continuous renal replacement therapy in the management of acute kidney injury associated with sinusoidal obstruction syndrome following hematopoietic cell transplantation. Pediatr Transplant. (2018) 22(5):481-7. doi: 10.1111/petr.13139, PMID: 29388370

[45] Goldstein SL. Fluid management in acute kidney injury. J Intensive Care Med. (2014) 29:183–9. doi: 10.1177/0885066612465816, PMID: 23753221

[46] Khoury HJ Wang T Hemmer MT Couriel D Alousi A Cutler C. Improved survival after acute graft-versus-host disease diagnosis in the modern era. Haematologica. (2017) 102:958–66. doi: 10.3324/haematol.2016.156356, PMID: 28302712 PMC5477615

[47] Moffet JR Mahadeo KM McArthur J Hsing DD Gertz SJ Smith LS. Acute respiratory failure and the kinetics of neutrophil recovery in pediatric hematopoietic cell transplantation: a multicenter study. Bone Marrow Transplant. (2020) 55:341–8. doi: 10.1038/s41409-019-0649-3, PMID: 31527817 PMC7091821

[48] Contejean A Lemiale V Resche-Rigon M Mokart D Pène F Kouatchet A. Increased mortality in hematological Malignancy patients with acute respiratory failure from undetermined etiology: a Groupe de Recherche en Réanimation Respiratoire en Onco-Hématologique (Grrr-OH) study. Ann Intensive Care. (2016) 6:102. doi: 10.1186/s13613-016-0202-0, PMID: 27783381 PMC5080277

