# Supplementary material for: Endotheliopathy syndromes, TA-TMA, and SOS, are risk factors for morbidity and mortality in critically ill pediatric hematopoietic cell transplant recipients
Source: Front Oncol. 2025 Sep 3;15:1642939. doi: 10.3389/fonc.2025.1642939 (PMC12440924; doi:10.3389/fonc.2025.1642939)
Supplement: Supplementary Table 1 — Risk factors for first IMV extubation failure. [file DataSheet1.docx]

**Supplemental Table 1: Risk Factors for First IMV Extubation Failure**

| **Characteristic** | **IMV Success**, N = 25 | **IMV Extubation Failure**, N = 15 | **OR (95% CI)** | **P-value** |
| --- | --- | --- | --- | --- |
| Age at Admission | 3.5 (1.8, 8.9) | 3.8 (1.2, 15.9) | 1.06 (0.95, 1.19) | 0.27 |
| SOS | 11 (44%) | 8 (53%) | 1.45 (0.40, 5.40) | 0.57 |
| Grade 3-4 acute GVHD^ | 10 (40%) | 7 (47%) | 1.17 (0.29, 4.85) | 0.83 |
| TA- TMA | 15 (60%) | 8 (53%) | 0.76 (0.21, 2.81) | 0.68 |
| RRT | 11 (44%) | 10 (67%) | 2.55 (0.69, 10.3) | 0.17 |
| Infection | 20 (80%) | 10 (67%) | 0.5 (0.11, 2.18) | 0.35 |
| Relapse | 5 (20%) | 4 (27%) | 1.45 (0.31, 6.66) | 0.63 |
| Graft Failure | 0 (0%) | 0 (0%) | --* |  |
| HCT Day at Admission | 61 (25, 94) | 52 (12, 112) | 1 (0.99, 1.01) | 0.89 |
| PICU LOS (days) |  |  |  |  |
| <5 Days | 8 (32%) | 4 (27%) | Ref |  |
| >=14 Days | 12 (48%) | 8 (53%) | 1.33 (0.30, 6.40) | 0.71 |
| 5-14 Days | 5 (20%) | 3 (20%) | 1.20 (0.17, 7.97) | 0.85 |
| PICU Indication* |  |  |  |  |
| Hypotension | 2 (8.0%) | 1 (6.7%) |  |  |
| Increased WOB/Hypoxia | 17 (68%) | 12 (80%) |  |  |
| Neurologic (AMS, Seizure) | 2 (8.0%) | 2 (13%) |  |  |
| Other | 3 (12%) | 0 (0%) |  |  |
| Procedural | 1 (4.0%) | 0 (0%) |  |  |
| HFNC | 10 (40%) | 10 (67%) | 3 (0.81, 12.2) | 0.11 |
| NIPPV | 5 (20%) | 5 (33%) | 2 (0.46, 8.86) | 0.35 |
| Weight change (category) |  |  |  |  |
| < -5% | 0 (0%) | 0 (0%) | --* |  |
| -5% to 0% (0% not included) | 5 (20%) | 3 (20%) | 0.69 (0.11, 3.90) | 0.67 |
| 0% to 5% (5% not included) | 8 (32%) | 7 (47%) | Ref |  |
| ≥ 5% | 12 (48%) | 5 (33%) | 0.48 (0.11, 2.01) | 0.32 |
| Transplant Indications* |  |  |  |  |
| Hematologic malignancy | 11 (44%) | 10 (67%) |  |  |
| Hematologic non-malignancy | 4 (16%) | 0 (0%) |  |  |
| Immunologic | 8 (32%) | 4 (27%) |  |  |
| Metabolic | 2 (8.0%) | 1 (6.7%) |  |  |
| Respiratory Failure Etiology* |  |  |  |  |
| Lung Disease | 10 (40%) | 7 (47%) |  |  |
| Neuro (AMS/seizures) | 3 (12%) | 1 (6.7%) |  |  |
| Other | 2 (8.0%) | 0 (0%) |  |  |
| Procedural | 4 (16%) | 3 (20%) |  |  |
| Sepsis | 1 (4.0%) | 2 (13%) |  |  |
| Upper Airway Obstruction (including Mucostis) | 5 (20%) | 2 (13%) |  |  |
| GCS on Arrival* |  |  |  |  |
| <=6 | 6 (24%) | 2 (13%) |  |  |
| 7 to 9 | 2 (8.0%) | 1 (6.7%) |  |  |
| 10 to 12 | 1 (4.0%) | 1 (6.7%) |  |  |
| 13 to 14 | 3 (12%) | 4 (27%) |  |  |
| 15 | 13 (52%) | 7 (47%) |  |  |
| HFNC Duration (days) | 13 (9, 15) | 23 (2, 36) | 1.00 (1.00, 1.00) | 0.75 |
| NIPPV Duration (days) | 4 (2, 6) | 29 (21, 71) | 1.24 (0.87, 1.75) | 0.23 |
| Had neutrophil engraftment by PICU admission | 20 (80%) | 10 (67%) | 0.50 (0.11, 2.18) | 0.35 |

Abbreviations: sinusoidal obstruction syndrome (SOS), transplant associated thrombotic microangiopathy (TA-TMA), graft versus host disease (GVHD), peripheral blood stem cell (PBSC), renal replacement therapy (RRT), length of stay (LOS), pediatric intensive care unit (PICU), work of breathing (WOB), altered mental status (AMS), high flow nasal cannula (HFNC), non-invasive positive pressure ventilation (NIPPV), ^maximum acute GVHD grade

**Supplemental Table 2: Outcomes of patients requiring renal replacement therapy by weight gain at time of RRT**

| Weight Change Category | N | Death while on RRT, n | Death after RRT, n | Alive at last follow-up, n | SOS | TA-TMA |
| --- | --- | --- | --- | --- | --- | --- |
| <5% | 11 | 8 (73%) | 2 (18%) | 1 (9%) |  |  |
| 5-10% | 7 | 1 (14%) | 5 (71%) | 1 (14%) |  |  |
| >10% | 5 | 1 (25%) | 3 (75%) | 0 (0%) |  |  |

**Supplemental Table 3:** Causes of Death (n=32)

|  | n, % |
| --- | --- |
| Primary COD | |
| Relapsed Disease | 11 (35.5) |
| Organ Failure | 9 (28.1) |
| Acute GVHD | 7 (21.9) |
| Infection | 5 (15.6) |
| Contributing | |
| Infection | 15 (46.9) |
| Organ failure | 8 (25) |
| TA-TMA present at death | |
| Yes | 20 (62.5) |
| SOS present at death | 4 (12.5) |

**Supplemental Figure 1: Infections of HCT patients admitted to the ICU**

Among the 56 patients and 91 ICU admissions, 66 admissions were complicated by ≥ 1 identified infection. Many patients had more than one organism/ infection and site involved. Viral infections were the most common (n=54) with most detected in the blood (n=26), nasopharyngeal swab (n=14), and lungs via bronchoscopy (n=3). Among bacterial infections (n=40), the majority were detected in the blood (n=27), gut (n=4), and lungs (n=3). Fungal infections were the least common, though aspergillus was identified the lungs (n=3), CNS (n=1), and Candida in the blood (n=2).

**Supplemental Figure 2**: Overall survival in HCT patients admitted to the ICU who had a cardiac arrest.


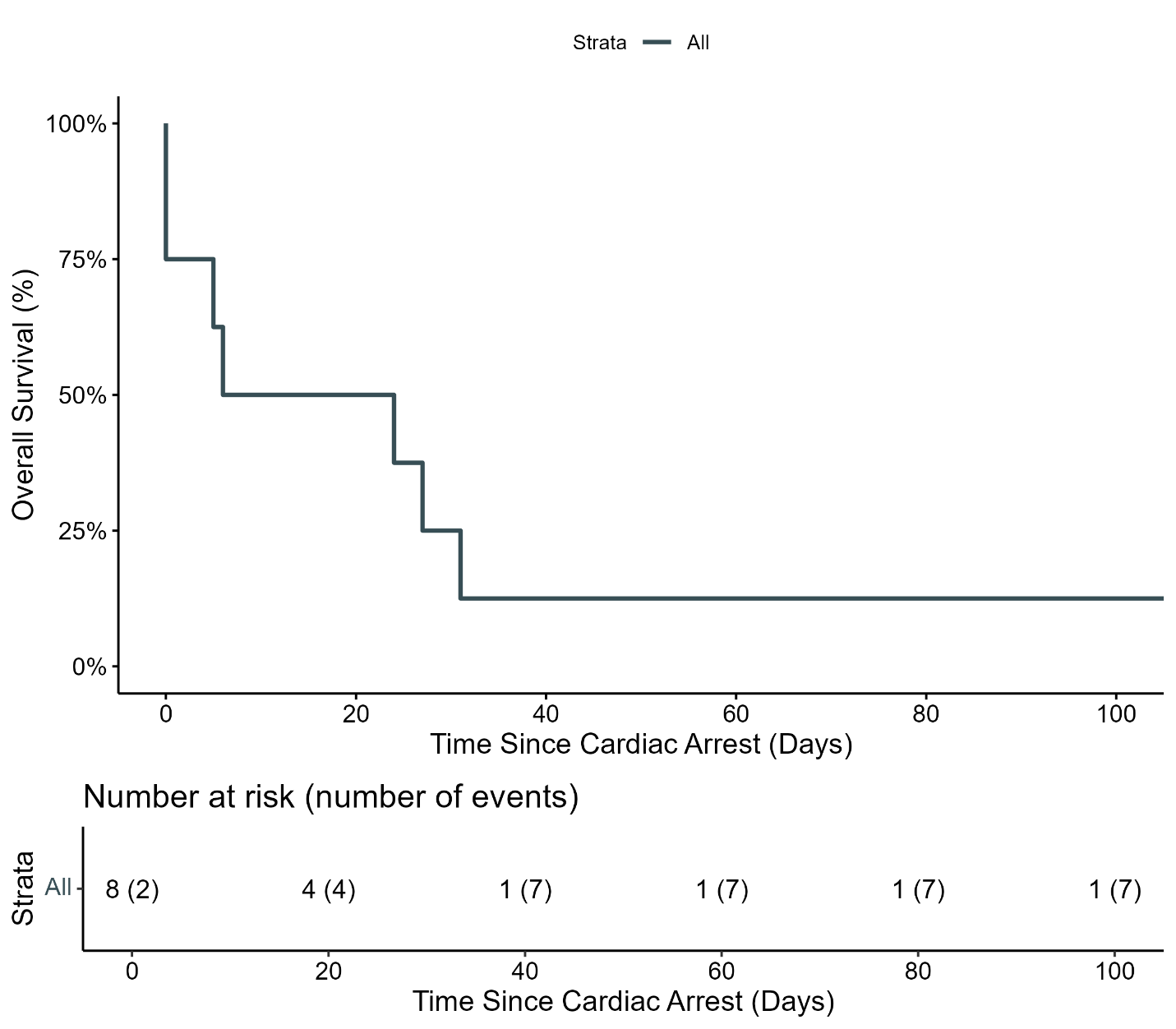


The estimated 100 day OS from the first cardiac arrest was 12.5% (95% CI 2.0% to 78.2%).

**Supplemental Figure 3. PICU Non-Relapse Mortality**

**
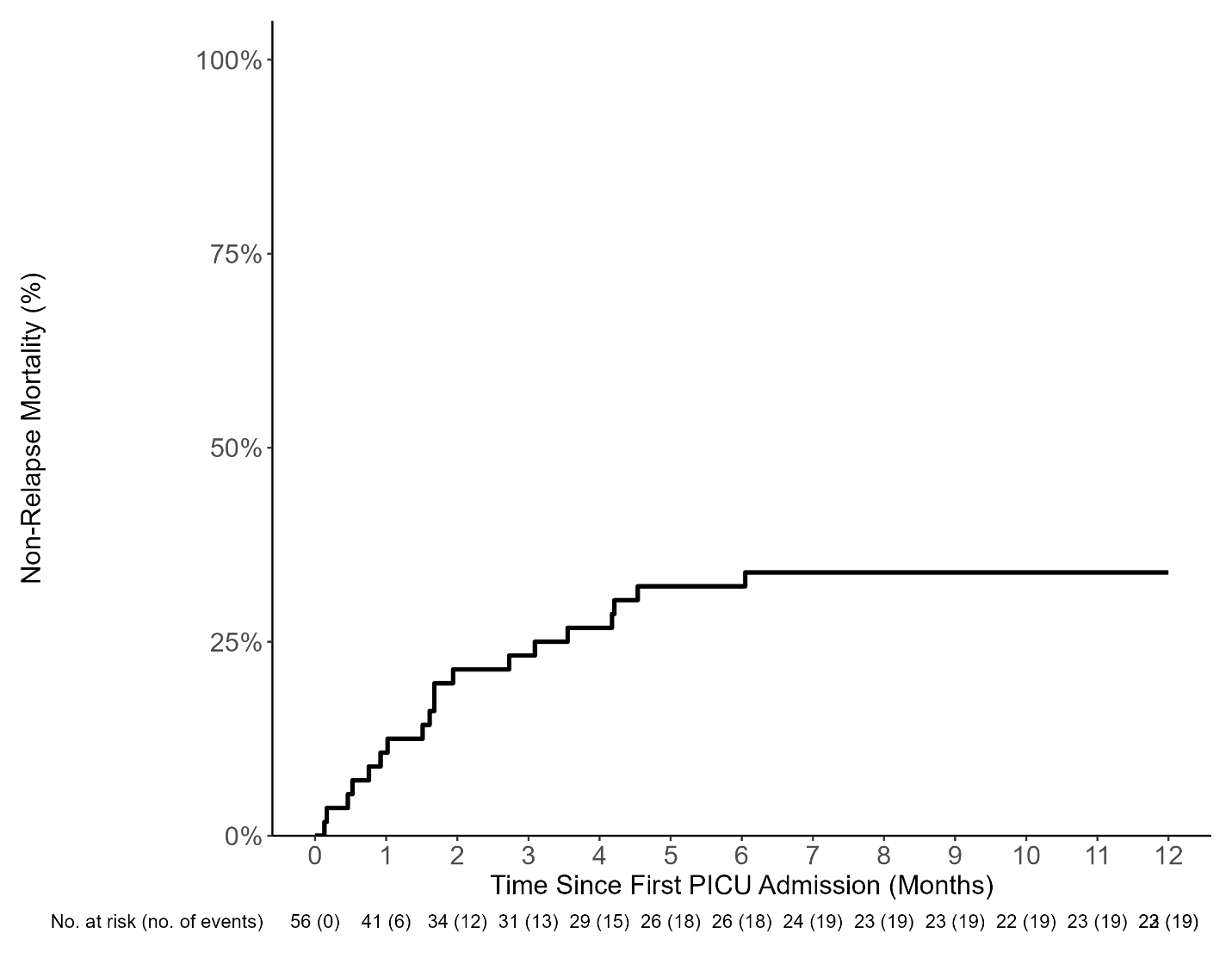
**

The estimated 100 day PICU mortality (death during first PICU admission from any cause other than relapse) from day of PICU admissions was 25% (95% 14%, 37%). Relapse was treated was a competing risk.
